# A Large‐Scale, Retrospective Analysis of Bath‐Psoralen Plus Ultraviolet A Therapy for Psoriasis: A Single‐Center Study

**DOI:** 10.1111/phpp.70038

**Published:** 2025-08-13

**Authors:** Mai Sakurai, Yuki Enomoto, Yoshifumi Kanayama, Takashi Sakaida, Aya Yamamoto, Akimichi Morita

**Affiliations:** ^1^ Department of Geriatric and Environmental Dermatology Nagoya City University Graduate School of Medical Sciences Nagoya Japan

**Keywords:** bath‐PUVA (bath‐psoralen plus ultraviolet a therapy), phototherapy, psoriasis

## Abstract

**Background/Purpose:**

While biologics and small‐molecule inhibitors are first‐line systemic treatments for psoriasis, phototherapy remains an alternative for patients unable to access these treatments because of medical or financial constraints. Narrow‐band ultraviolet B (NB‐UVB) is effective for localized psoriasis but less so for extensive disease. To address this limitation, bathwater delivery of psoralen plus ultraviolet A (bath‐PUVA) was introduced in 2004. This study evaluates the efficacy, safety, and patient characteristics associated with bath‐PUVA therapy in a large cohort.

**Methods:**

This retrospective analysis included 229 patients (180 males, 49 females) treated with bath‐PUVA from 2004 to September 2021. Baseline characteristics and treatment outcomes were assessed using the psoriasis area and severity index (PASI). Statistical analyses examined relationships between treatment outcomes and factors, including baseline PASI, body mass index (BMI), and smoking status.

**Results:**

The mean baseline PASI score was 24.9. Bath‐PUVA achieved PASI 75 in 80.4% of patients, PASI 90 in 44.1%, and PASI 100 in 2.6%, with efficacy comparable to biologics. Patients achieving PASI 90 had significantly higher baseline PASI scores (*p* = 0.005), while the number of irradiations required did not differ (*p* = 0.692). Higher baseline PASI scores correlated with elevated BMI (*p* = 0.002), but BMI did not influence improvement rates (*p* = 0.094). Smokers had significantly higher baseline PASI scores (*p* = 0.004) compared with non‐smokers, yet smoking status did not affect improvement rates (*p* = 0.862).

**Conclusion:**

Bath‐PUVA demonstrates efficacy comparable with biologics for psoriasis, regardless of BMI or smoking status. This analysis supports its use as an effective and accessible treatment option for patients with extensive disease.

## 1 Introduction

Psoriasis is a chronic inflammatory skin disorder influenced by genetic predisposition and environmental factors such as smoking, obesity, medications, and infections. These triggers activate the skin's immune system, resulting in abnormal epidermal cell proliferation and differentiation. A global systematic review reported prevalence rates of 0.51% to 11.4% in adults and 0% to 1.37% in children [1]. Prevalence increases with distance from the equator, suggesting a potential protective role of ultraviolet radiation. Psoriasis is classified into four subtypes: (i) chronic plaque psoriasis (CPP), comprising 90% of cases; (ii) erythrodermic psoriasis (EP), affecting over 90% of the body surface; (iii) generalized pustular psoriasis (GPP), with widespread pustules; and (iv) guttate psoriasis (GP), an acute form presenting with small papules (< 1 cm) often triggered by infections in children and young adults [2]. Psoriasis is increasingly recognized as a systemic inflammatory disease associated with comorbidities, including psoriatic arthritis (PsA), metabolic syndrome (MS) components (e.g., obesity, hypertension, dyslipidemia, diabetes mellitus), atherosclerosis, chronic obstructive pulmonary disease (COPD), chronic kidney disease (CKD), non‐alcoholic fatty liver disease (NAFLD), uveitis, psychiatric disorders (e.g., depression), autoimmune diseases, malignancies, and inflammatory bowel disease [3, 4, 5]. Approximately 70%–80% of patients have mild disease manageable with topical therapies alone [6]. Treatment for moderate‐to‐severe psoriasis includes ultraviolet phototherapy, systemic oral therapies, and biologics. When biologics are contraindicated or declined, ultraviolet phototherapy offers a practical alternative.

Narrow‐band ultraviolet B (NB‐UVB) phototherapy is widely employed in clinical practice. Comparative studies of psoralen plus ultraviolet A (PUVA) therapy and NB‐UVB for CPP show PUVA achieves psoriasis area and severity index (PASI) 75% in 80% of cases versus 70% with NB‐UVB [7]. A meta‐analysis found PUVA more likely to achieve remission at 6 months than NB‐UVB [odds ratio (OR) = 2.73; 95% confidence interval (CI) 1.19–6.27, *p* = 0.02] and required fewer sessions for clearance (17 vs. 25). Although PUVA offers greater efficacy and longer remission, NB‐UVB is preferred for its convenience, absence of photosensitizers, and lower erythema risk. PUVA is reserved for NB‐UVB‐resistant cases or rapid relapse [8]. Bathwater delivery of PUVA (bath‐PUVA) therapy is an alternative but is available at limited facilities. No retrospective studies in Japan have assessed bath‐PUVA outcomes in patients with and without comorbidities. This study aimed to address these gaps by examining the therapeutic efficacy and safety of bath‐PUVA therapy.

## 2 Materials and Methods

### 2.1 Participants

This single‐center, retrospective cohort study included clinical data from patients diagnosed with psoriasis at Nagoya City University Hospital between January 2004 and September 2021 (*n* = 229). Patients included in the analysis had undergone bath‐PUVA therapy during this period. Psoriasis subtypes among participants comprised CPP (*n* = 195), EP (*n* = 16), GPP (*n* = 17), and GP (*n* = 1). The cohort included patients who had previously received treatments such as cyclosporine, biologics, or ultraviolet phototherapy (e.g., NB‐UVB, outpatient bath‐PUVA, or topical PUVA) at other institutions. Ethical approval was obtained from the Ethics Review Board of Nagoya City University (#60‐23‐0096), and the study adhered to the Declaration of Helsinki and Ethical Guidelines for Clinical Research.

### 2.2 Bath‐PUVA Protocol

Bath‐PUVA therapy involved immersion in water at 37°C containing 8‐methoxypsoralen bath salts at a concentration of 0.0001% for 15 min, followed by immediate ultraviolet A (UVA) irradiation. Phototherapy was conducted using the upright UV‐7002A device five times weekly. From January 2004 to May 2005, UVA doses followed an incremental schedule (0.2, 0.5, 0.8, 1.1, 1.5, 1.9, 2.3, 2.8, 3.3, and 4.0 J/cm^2^), with a fixed dose of 4.0 J/cm^2^. After June 2005, the initial dose was set at 0.5 J/cm^2^, increasing incrementally by 0.5 J/cm^2^ until reaching 4.0 J/cm^2^, which was then maintained. Patients were discharged once lesion improvement was achieved. Relapse cases were managed through alternative treatments or readmission for resumption of UVA irradiation five times weekly.

### 2.3 Clinical Data Collection

The following clinical data were collected: sex, psoriasis subtype, age at disease onset, age at initiation of bath‐PUVA therapy, disease duration (time from onset to therapy initiation), body mass index (BMI), smoking history (self‐reported), pre‐ and post‐therapy PASI scores (including component scores), number of UVA sessions, comorbidity presence and types, prior treatments, subsequent treatment progression, and family history. For patients who underwent multiple treatments, data from the initial admission were analyzed.

### 2.4 Statistical Analysis

### 2.5 Statistical analyses were conducted using PharmacoBasic Software (Scientist Press co. Ltd., Tokyo, Japan). Comparative analyses included Student's *t*‐test for two‐group comparisons and the Williams and Tukey tests for comparisons among three or more groups, such as BMI and smoking history. Graphs were generated using GraphPad Prism (Version 10, GraphPad Software, San Diego, CA, USA)

## 3 Results

### 3.1 Patient Characteristics

The demographic and clinical characteristics of 229 psoriasis patients treated with bath‐PUVA therapy during hospitalization at Nagoya City University Hospital from January 2004 to September 2021 are presented in Table 1.

**TABLE 1:** Baseline characteristics and treatment details of patients with psoriasis.

Feature	
Sex, *n*(%)	
Male	180(78.6)
Female	49(21.4)
Clinical subtypes, *n*(%)	
Chronic plaque psoriasis	195(85.2)
Erythrodermic psoriasis	16(7.0)
Pustular psoriasis	17(7.4)
Guttate psoriasis	1(0.4)
Family history of psoriasis	9(3.9)
Age, mean ± SD, years	54.2 ± 16.3
BMI, mean ± SD, kg/m^2^	24.6 ± 4.4
Age of onset, mean ± SD, years	43.5 ± 18.1
Period until bath‐PUVA therapy, mean ± SD, years	10.7 ± 8.9
PASI at treatment initiation, mean ± SD	24.9 ± 12.6
PASI achievement, mean ± SD	84 ± 14.1
Absolute PASI after bath‐PUVA, mean ± SD	3.8 ± 4.2
Number of irradiations	21.6 ± 8.6
Number of admissions	1.9 ± 2.1
Smoking status, *n*(%)	
Current smoker	78(34.1)
Ex‐smoker	22(9.6)
Never‐smoker	96(41.9)
Unknown	33(14.4)
Prior treatment, *n*(%)	
Cyclosporine	10(4.4)
Biologics	2(0.9)
Phototherapy	
NB‐UVB	20(8.7))
Bath‐PUVA as outpatient Department	4(1.7)
Topical PUVA at other hospitals	2(0.9)
Combined treatment	
Etretinate	19(8.3)
Apremilast	1(0.4)

Abbreviations: bath‐PUVA, bathwater delivery of psoralen plus ultraviolet A; BMI, body mass index; NB‐UVB, narrow‐band ultraviolet B; PASI, psoriasis area and severity index; SD, standard deviation.

### 3.2 Treatment Efficacy

**FIGURE 1:**
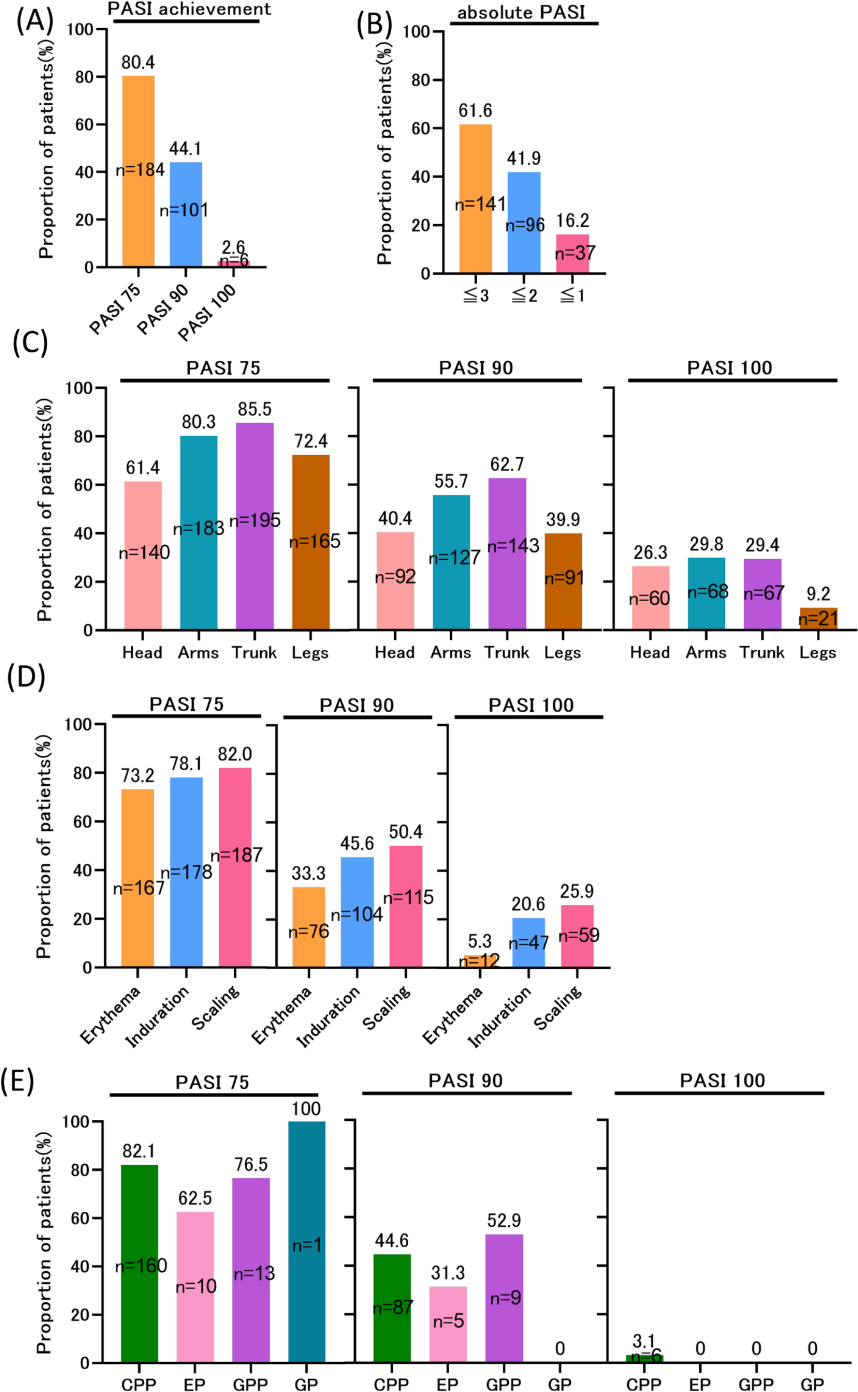
(A) Numbers and percentages of patients achieving PASI 75, PASI 90, and PASI 100, indicating PASI achievement. (B) Numbers and percentages of patients with absolute PASI scores of ≤ 3, ≤ 2, and ≤ 1. (C) Numbers and percentages of patients achieving PASI 75, PASI 90, and PASI 100 for each body component. (D) Numbers and percentages of patients achieving PASI 75, PASI 90, and PASI 100 for each PASI component (erythema, induration, and scaling). (E) Numbers and percentages of patients achieving PASI 75, PASI 90, and PASI 100 for each psoriasis subtype. CPP, chronic plaque psoriasis; EP, erythrodermic psoriasis; GP, guttate psoriasis; GPP, generalized pustular psoriasis; PASI, psoriasis area and severity index.

A total of 80.4% (184 patients) achieved PASI 75, 44.1% (101 patients) achieved PASI 90, and 2.6% (6 patients) achieved PASI 100. Absolute PASI scores post‐therapy indicated significant improvement, with 61.6% (141 patients) achieving PASI ≤ 3, 41.9% (96 patients) achieving PASI ≤ 2, and 16.2% (37 patients) achieving PASI ≤ 1. These reductions were comparable to those seen with biologic therapies (Figure 1A,B). A representative case achieving PASI 90 after 19 irradiations and a cumulative dose of 62 J/cm^2^ is shown in Figure S1.

No significant sex‐based differences were observed, with mean PASI improvement rates of 84% in both males and females. Post‐treatment mean PASI scores were 3.7 in males and 4.0 in females (*p* = 0.6). The number of irradiations and hospital admissions did not differ significantly between PASI 90 achievers and non‐achievers (*p* = 0.692 and *p* = 0.824, respectively). However, PASI 90 achievers had significantly higher baseline PASI scores than non‐achievers (*p* = 0.005), indicating that higher initial severity does not limit bath‐PUVA therapy efficacy.

Achievement rates for PASI 75, PASI 90, and PASI 100 varied by anatomical region. PASI 75 rates were 85.5% (195 patients) for the trunk, 80.3% (183 patients) for the upper limbs, 72.4% (165 patients) for the lower limbs, and 61.4% (140 patients) for the head. PASI 90 rates were 62.7% (143 patients), 55.7% (127 patients), 39.9% (91 patients), and 40.4% (92 patients), respectively, while PASI 100 rates were 29.4% (67 patients), 29.8% (68 patients), 9.2% (21 patients), and 26.3% (60 patients), respectively. Treatment effects followed the order: trunk>upper limbs>lower limbs>head (Figure 1C). Achieving PASI 100 was particularly challenging for the lower limbs, likely because of treatment difficulty in this area. In addition, due to the structural limitations of the irradiation cabinets, UVA exposure to the lower extremities may be reduced because of lower UVA output at the bottom of the devices. For the head, treatment involved applying towels soaked in the medicated solution. Examination of PASI components showed sequential improvement: scaling resolved first (PASI 75: 82.0%, PASI 90: 50.4%, PASI 100: 25.9%), followed by induration (PASI 75: 78.1%, PASI 90: 45.6%, PASI 100: 20.6%), and then erythema (PASI 75: 73.2%, PASI 90: 33.3%, PASI 100: 5.3%) (Figure 1D).

Efficacy also varied among psoriasis subtypes. PASI 75 rates were 82.1% (160 patients) for CPP, 62.5% (10 patients) for EP, 76.5% (13 patients) for GPP, and 100% (1 patient) for GP. PASI 90 rates were 44.6% (87 patients) for CPP, 31.3% (5 patients) for EP, and 52.9% (9 patients) for GPP. PASI 100 was achieved by 3.1% (6 patients) in CPP (Figure 1E). EP patients had a significantly lower mean PASI improvement rate (72.3%) compared with non‐erythrodermic subtypes (84.9%, *p* = 0.001) and required more irradiations (mean: 26.2 vs. 21.2; *p* = 0.026). Among EP patients, leaner individuals (mean BMI: 20.4 kg/m^2^) were more likely to achieve PASI 90 compared with those with higher BMI (mean BMI: 25.6 kg/m^2^; *p* = 0.034).

### 3.3 Disease Duration and PASI Improvement Rate

Disease duration, defined as the time from psoriasis onset to the initiation of inpatient bath‐PUVA therapy, had a mean value of 10.7 years, with a maximum duration of 49 years. Patients with a short disease duration (≤ 2 years, *n* = 40) exhibited a mean PASI improvement rate of 81.0%. This rate was not significantly different (*p* = 0.142) from the mean improvement rate of 84.6% observed in patients with disease duration ≥ 3 years (*n* = 189). These findings indicate that bath‐PUVA therapy efficacy is independent of disease duration.

### 3.4 Association Between BMI and PASI Outcomes

**FIGURE 2:**
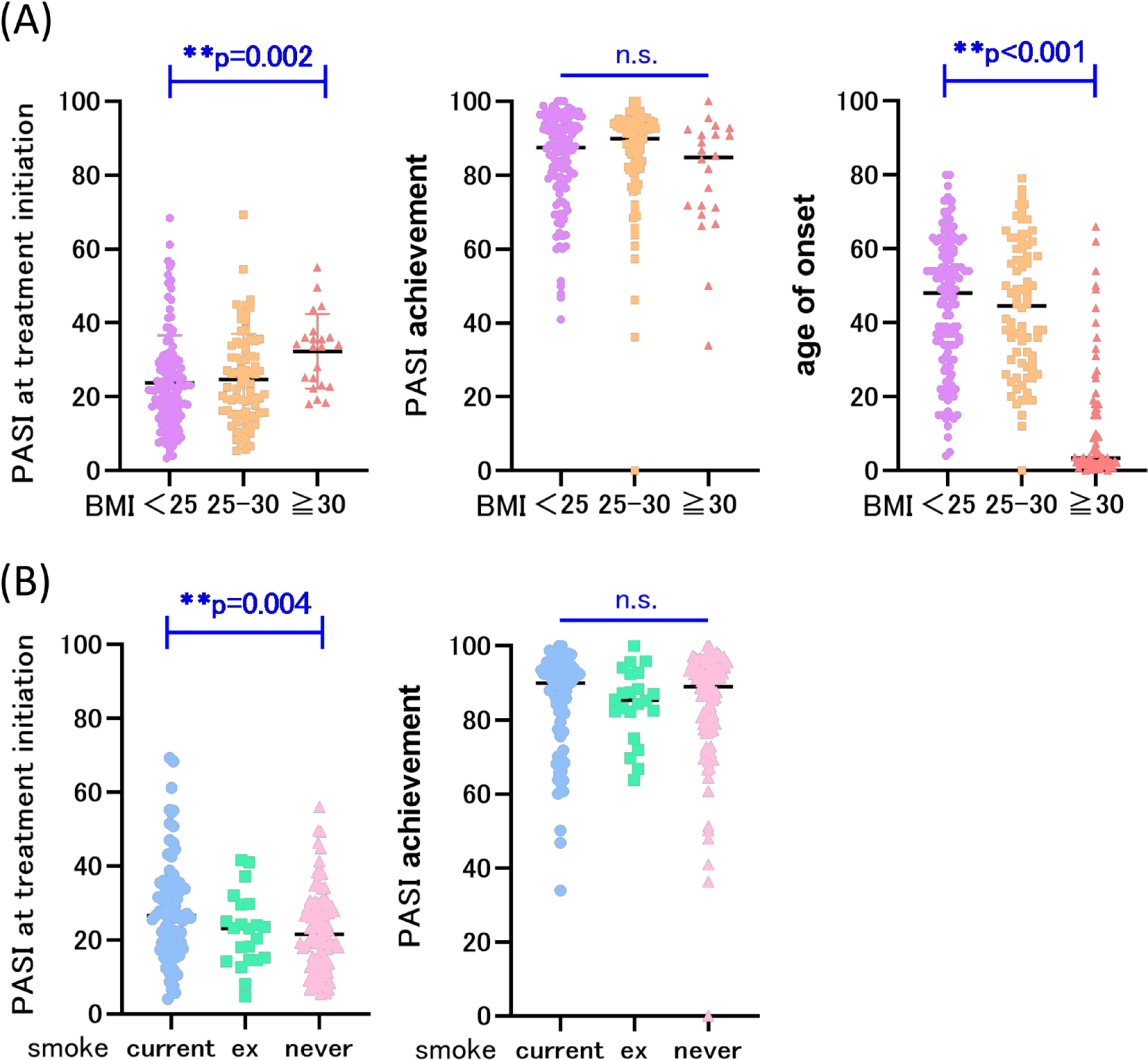
(A) BMI and PASI score at hospital admission, PASI improvement rate, and BMI and age of onset. (B) Smoking history, PASI score at hospital admission, and PASI improvement rate. BMI, body mass index; n.s., not significant; PASI, psoriasis area and severity index.

Patients were stratified into three BMI groups: BMI < 25 (*n* = 129), 25 ≤ BMI < 30 (*n* = 78), and BMI ≥ 30 kg/m^2^ (*n* = 22). The mean PASI scores at hospital admission were 23.8, 24.7, and 32.3, respectively. There was no significant difference in admission PASI scores between the BMI < 25 and 25 ≤ BMI < 30 groups (*p* = 0.433); however, patients with BMI ≥ 30 kg/m^2^ had significantly higher PASI scores compared with both groups (Williams test, *p* = 0.002). The mean PASI improvement rates were 84.2%, 85.1%, and 80.2% for the BMI < 25, 25 ≤ BMI < 30, and BMI ≥ 30 kg/m^2^ groups, respectively. BMI did not significantly influence PASI improvement rates (Williams test, *p* = 0.094), indicating that bath‐PUVA efficacy is not BMI‐dependent. Additionally, the mean age at psoriasis onset was 45.1 years for BMI < 25, 43.9 years for 25 ≤ BMI < 30, and 11.7 years for BMI ≥ 30 kg/m^2^. There was no significant difference in age of onset between the BMI < 25 and 25 ≤ BMI < 30 groups (*p* = 0.485); however, patients with BMI ≥ 30 kg/m^2^ had a significantly younger onset age (*p* < 0.001) (Figure 2A).

### 3.5 Association Between Smoking History and PASI Outcomes

Smoking history was categorized into current smokers (*n* = 78), ex‐smokers (*n* = 22), and never‐smokers (*n* = 96), with smoking status unknown for 33 patients. Current smokers had significantly higher PASI scores at hospital admission compared with never‐smokers (Tukey test, *p* = 0.004). No significant differences were observed between never‐smokers and ex‐smokers or between ex‐smokers and current smokers. The mean PASI improvement rates were 84.5%, 84.2%, and 84.1% for current smokers, ex‐smokers, and never‐smokers, respectively. No significant differences in PASI improvement rates were identified between these groups (Tukey test). Therefore, the efficacy of bath‐PUVA therapy appears to be unaffected by smoking history (Figure 2B).

### 3.6 Association With Comorbidities (Table 2, Figure 3, Figure S2)

**TABLE 2:** Prevalence of comorbidities among study participants.

Comorbidities, *n* (%)	
Psoriatic arthritis	36(15.7)
Obesity	22(9.6)
High blood pressure	61(26.6)
High cholesterol	31(13.5)
Diabetes mellitus	47(20.5)
Atherosclerotic disease	18(7.9)
Chronic kidney disease	17(7.4)
Non‐alcoholic fatty liver disease	5(2.2)
Chronic obstructive pulmonary disease	11(4.8)
Psychiatric disorder	15(6.6)
Uveitis	2(0.9)
Autoimmune diseases	19(8.3)
Malignant tumor	33(14.4)

**FIGURE 3:**
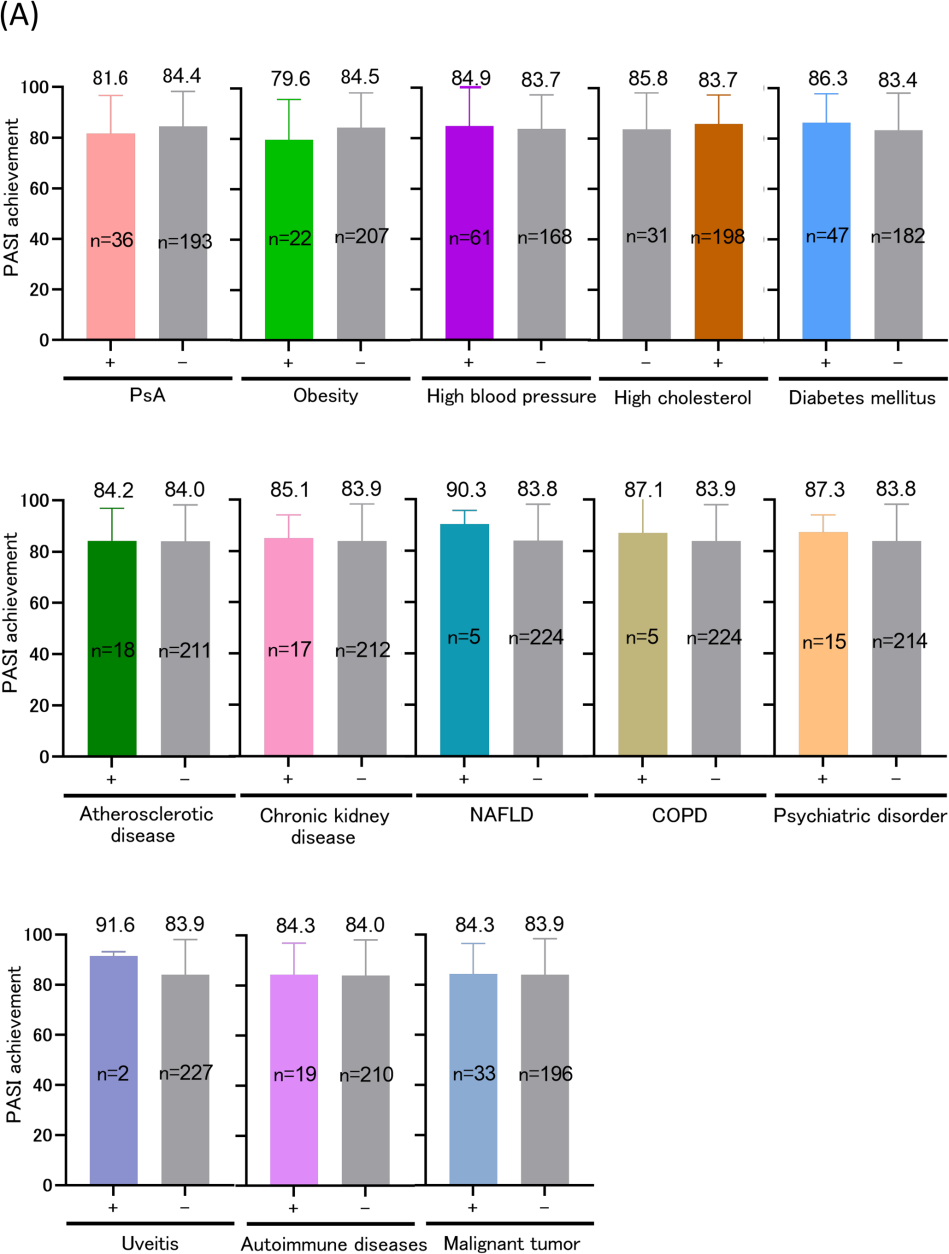
(A) Presence or absence of comorbidities and PASI improvement rate. COPD, chronic obstructive pulmonary disease; NAFLD, non‐alcoholic fatty liver disease; PASI, psoriasis area and severity index; PsA, psoriatic arthritis.

Table 2 summarizes the comorbidities and patient counts in the study. CKD included four patients undergoing dialysis. Autoimmune diseases included Hashimoto's disease, Graves' disease, rheumatoid arthritis, bullous pemphigoid, anti‐laminin γ1 pemphigoid, and IgA nephropathy. Malignancies included prostate cancer, colorectal cancer, malignant lymphoma, and myelodysplastic syndrome, with some patients having multiple cancers. Inflammatory bowel disease was absent in this cohort. PASI improvement rates, stratified by the presence or absence of psoriasis‐associated comorbidities, are presented in Figure 3. No significant differences in PASI improvement rates were observed for comorbidities such as PsA, obesity, hypertension, hypercholesterolemia, diabetes mellitus, atherosclerotic diseases, CKD, NAFLD, COPD, psychiatric disorders, uveitis, autoimmune diseases, or malignancies. Bath‐PUVA therapy demonstrated comparable PASI 75, PASI 90, and PASI 100 achievement rates across all comorbidity groups (Figure S2).

Regarding comorbidities and disease severity, patients with PsA, obesity (BMI ≥ 30 kg/m^2^), and atherosclerotic diseases (ischemic heart disease and cerebral infarction) exhibited significantly higher PASI scores at admission (PsA: *p* = 0.037, obesity: *p* = 0.004, atherosclerotic diseases: *p* = 0.008). PsA was associated with more irradiation sessions (*p* = 0.001) and persistently higher absolute PASI values (*p* = 0.004), although PASI improvement rates were unaffected. Obesity correlated with higher absolute PASI values (*p* < 0.001) but did not significantly affect irradiation sessions or PASI improvement rates. Diabetes mellitus had no significant impact on PASI scores at admission, absolute PASI, or improvement rates but was associated with fewer irradiation sessions (*p* = 0.027). Other comorbidities showed no significant differences in PASI scores at admission, absolute PASI, number of hospital admissions, or irradiation sessions.

### 3.7 Previous Treatment

Two patients with prior biologic exposure had a history of infliximab administration. In the GPP patient, infliximab was ineffective and discontinued, leading to bath‐PUVA therapy initiation after 2 months. Similarly, the CPP patient also found infliximab ineffective, starting inpatient bath‐PUVA therapy after 14 months.

Among the 229 patients, concurrent oral medications during bath‐PUVA therapy included etretinate in 19 (8.3%) and apremilast in one (0.4%). These were either continued from prior prescriptions or introduced as a result of suboptimal bath‐PUVA progress. Patients receiving concurrent medications had significantly higher PASI scores at admission (mean: 31.1) than those without (mean: 24.3; *p* = 0.021). The PASI improvement rate was higher in the no‐concurrent‐medication group (77.5%; *p* = 0.032). Furthermore, the no‐concurrent‐medication group required fewer phototherapy sessions (mean: 21.1) compared with the concurrent‐medication group (mean: 26.5; *p* = 0.008).

### 3.8 Post‐Hospital Admission Outcomes for Bath‐PUVA Therapy

The outcomes of bath‐PUVA therapy following hospital admission were analyzed for 229 patients. A single admission sufficed for 143 patients (62.4%), with others requiring up to 21 admissions. Of these 143 patients, 94 (65.7%) managed without subsequent biologics or molecular‐targeted therapies, were reverse‐referred, or discontinued outpatient follow‐ups, demonstrating the efficacy of one inpatient regimen. Patients averaged 21.6 phototherapy sessions per admission.

As of December 2022, treatment outcomes were as follows: 128 patients (55.9%) discontinued treatment, were referred to other institutions, or continued topical therapies alone; 69 (30.1%) used biologics; and 29 (12.7%) were on oral systemic medications, including etretinate, apremilast, deucravacitinib, methotrexate, cyclosporine, and prednisolone. Two patients continued outpatient phototherapy (bath‐PUVA or NB‐UVB), and one received combined phototherapy and etretinate. Among the 69 patients on biologics, agents included anti‐TNF‐α inhibitors (31, 44.9%), anti‐IL‐17 antibodies (13, 18.8%), anti‐IL‐23 antibodies (19, 27.5%), and anti‐IL12/23 p40 antibodies (6, 8.7%).

### 3.9 Safety

None of the patients exhibited any severe (grade 3 or higher) adverse events during bath‐PUVA therapy, and no cases required treatment discontinuation due to adverse events.

## 4 Discussion

Biologics and small‐molecule agents are first‐line treatments for moderate‐to‐severe psoriasis when conventional systemic therapies are ineffective or intolerable. Although effective, phototherapy remains a viable alternative for patients unable to use biologics because of medical, financial, or personal constraints. Among phototherapies, NB‐UVB is the most widely employed given its ease of use and favorable safety profile; it does not require the photosensitizer psoralen used in PUVA therapy or post‐treatment light avoidance. Additionally, NB‐UVB lacks a short‐wavelength component, reducing the risk of erythema and improving therapeutic outcomes. For patients with high PASI scores or inadequate NB‐UVB response, bath‐PUVA therapy offers an effective alternative. A large‐scale retrospective analysis at our institution found bath‐PUVA therapy achieved efficacy comparable with certain biologics, with outcomes independent of BMI, smoking status, or comorbidities.

Consistent with an Italian study [9], substantial PASI score reductions and good response rates were observed with bath‐PUVA. At our institution, baseline PASI scores were higher, yet similar levels of improvement were achieved, emphasizing the robustness of bath‐PUVA therapy. Adverse effects were mild and transient, aligning with previous reports.

The influence of comorbidities on phototherapy outcomes has been explored in previous research. One study [10] demonstrated that patients with metabolic syndrome (MS) undergoing NB‐UVB for CPP experienced significantly lower PASI improvements compared with those without MS, identifying MS as an independent predictor of diminished efficacy. In contrast, bath‐PUVA therapy at our institution showed consistent efficacy regardless of comorbidities, with no significant differences in PASI improvement rates. These findings reinforce the suitability of bath‐PUVA therapy for a diverse range of patients, including those with complex medical histories.

Regarding disease duration, shorter durations have been associated with better outcomes in biologic therapies [11]. However, our findings indicate that the efficacy of bath‐PUVA therapy is not influenced by the time from psoriasis onset to therapy initiation. This suggests bath‐PUVA therapy has broad applicability across different stages of disease progression.

Concurrent medications have also been shown to affect phototherapy outcomes. Studies have reported that combining etretinate with PUVA reduces the total irradiation dose required while maintaining similar PASI 75 rates compared with PUVA alone [12]. Other research [13] have found that combining apremilast with phototherapy does not significantly alter PASI 75 rates but allows many patients to achieve PASI 75 within 8 weeks. At our institution, patients on combination therapies presented with higher baseline PASI scores, reflecting their use in severe cases. Although PASI improvement rates were higher without concurrent therapies, combination treatments required more irradiation sessions, highlighting the need for tailored strategies in severe cases.

Long‐term outcomes favor bath‐PUVA therapy, with many patients achieving sustained remission without requiring biologics, molecular‐targeted drugs, or frequent follow‐ups. Studies comparing NB‐UVB and oral PUVA found superior remission rates with PUVA [14], and our findings affirm the potential of bath‐PUVA for durable disease control.

Biologics, despite their efficacy, pose significant cost barriers. In Japan, achieving PASI 75 with any of 11 approved biologics incurs substantial expense. A systematic review [15] of 22 US‐based economic studies (2008–2013) estimated annual psoriasis‐related direct costs at $51.7–$63.2 billion and indirect costs at $23.9–$35.4 billion, with comorbidities adding $36.4 billion. The lifetime cost was $11,498 per patient for relief of physical symptoms and emotional health; however, intangible cost data are limited. Among 114,512 psoriasis patients, 51% had at least one comorbidity, increasing emergency care use [OR (95% CI) = 1.58 (1.51–1.65)], hospitalization rates [incidence rate ratio (IRR) (95% CI) = 2.27 (2.13–2.42)], and outpatient visits [IRR (95% CI) = 1.53 (1.52–1.55)]. Comorbidities raised total costs by $2184 (*p* < 0.001) [16]. Bath‐PUVA therapy, effective across BMI, smoking status, and comorbidities, is particularly valuable in regions with high infectious disease prevalence (e.g., tuberculosis, HIV, hepatitis B) and limited resources.

In conclusion, bath‐PUVA therapy offers a safe, effective, and cost‐efficient option for moderate‐to‐severe psoriasis. Its affordability, durability, and broad applicability make it invaluable, particularly in resource‐limited settings, warranting further integration and optimization.

## Author Contributions

**Yuki Enomoto:** conceptualization; data curation; formal analysis; investigation; visualization; writing – review and editing. **Yoshifumi Kanayama:** investigation; writing – review and editing. **Takashi Sakaida:** data curation; investigation; writing – review and editing. **Mai Sakurai:** investigation; writing – review and editing. **Aya Yamamoto:** investigation; writing – review and editing. **Akimichi Morita:** conceptualization; project administration; supervision; writing – review and editing.

## Conflicts of Interest

The authors declare no conflicts of interest.

## Supporting information


**Figure S1.** Progress of a patient who received inpatient bath‐PUVA therapy. From left to right: At the time of admission, after 11 sessions (total dose: 30 J/cm^2^), after 15 sessions (total dose: 46 J/cm^2^), and after 19 sessions (total dose: 62 J/cm^2^). bath‐PUVA, bathwater delivery of psoralen plus ultraviolet A; PASI, psoriasis area and severity index.
**Figure S2.** Numbers and percentages of patients achieving PASI 75, PASI 90, and PASI 100, stratified by the presence or absence of comorbidities. COPD, chronic obstructive pulmonary disease; NAFLD, non‐alcoholic fatty liver disease; PASI, psoriasis area and severity index; PsA, psoriatic arthritis.

## Data Availability

The data that support the findings of this study are available from the corresponding author upon reasonable request.
