# Supplementary material for: A Large‐Scale, Retrospective Analysis of Bath‐Psoralen Plus Ultraviolet A Therapy for Psoriasis: A Single‐Center Study
Source: Photodermatol Photoimmunol Photomed. 2025 Aug 13;41(5):e70038. doi: 10.1111/phpp.70038 (PMC12350823; doi:10.1111/phpp.70038)
Supplement: Supplementary file 1 — Figure S1. Progress of a patient who received inpatient bath‐PUVA therapy. From left to right: At the time of admission, after 11 sessions (total dose: 30 J/cm2), after 15 sessions (total dose: 46 J/cm2), and after 19 sessions (total dose: 62 J/cm2). bath‐PUVA, bathwater delivery of psoralen plus ultraviolet A; PASI, psoriasis area and severity index. Figure S2. Numbers and percentages of patients achieving PASI 75, PASI 90, and PASI 100, stratified by the presence or absence of comorbidities. COPD, chronic obstructive pulmonary disease; NAFLD, non‐alcoholic fatty liver disease; PASI, psoriasis area and severity index; PsA, psoriatic arthritis. [file PHPP-41-e70038-s001.pptx]

## Slide 1
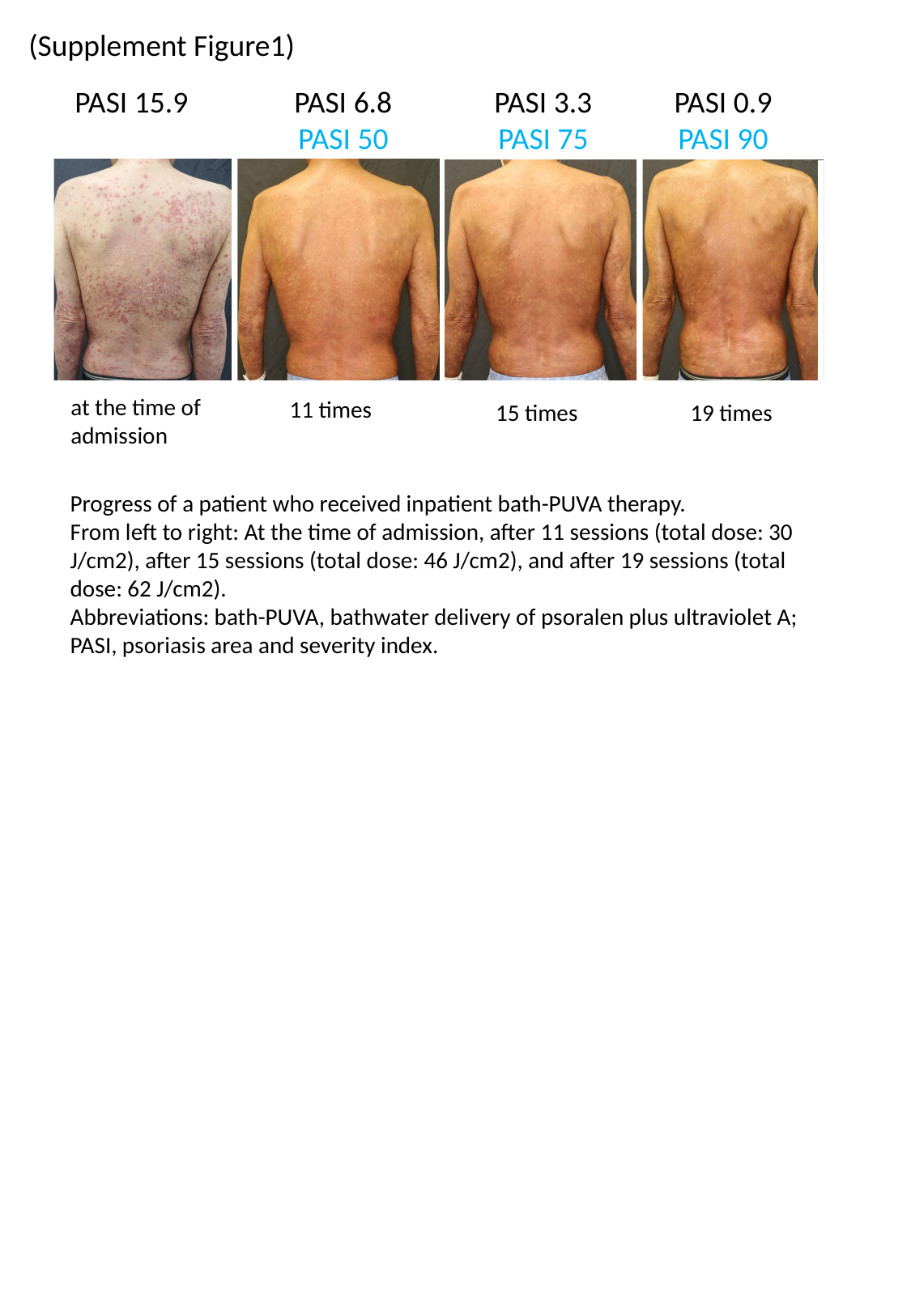

(Supplement Figure1)
PASI 15.9
PASI 6.8
PASI 50
PASI 3.3
PASI 75
PASI 0.9
PASI 90
　15 times
　19 times
11 times
at the time of admission
Progress of a patient who received inpatient bath-PUVA therapy.
From left to right: At the time of admission, after 11 sessions (total dose: 30 J/cm2), after 15 sessions (total dose: 46 J/cm2), and after 19 sessions (total dose: 62 J/cm2).
Abbreviations: bath-PUVA, bathwater delivery of psoralen plus ultraviolet A; PASI, psoriasis area and severity index.

## Slide 2
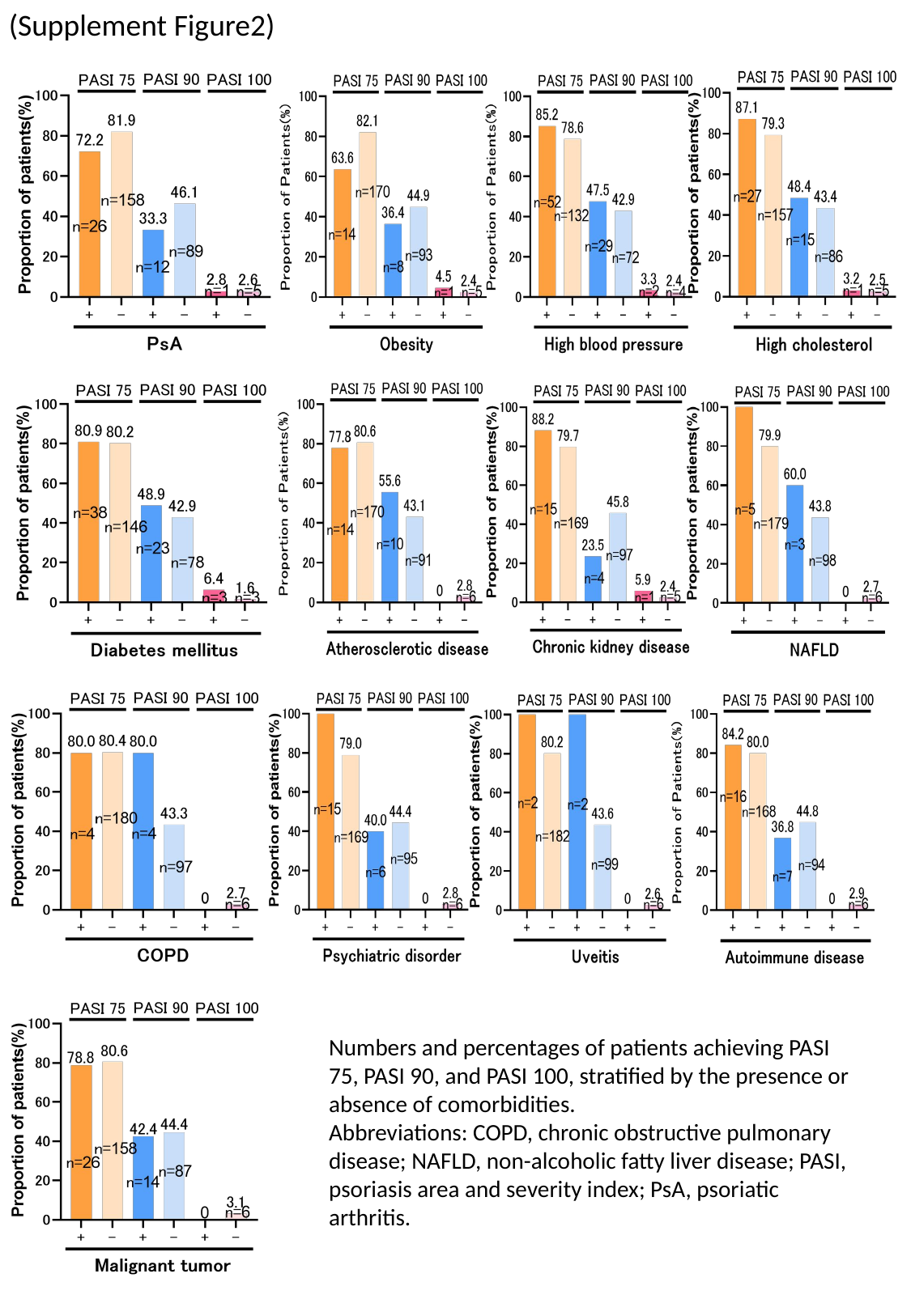

(Supplement Figure2)
Numbers and percentages of patients achieving PASI 75, PASI 90, and PASI 100, stratified by the presence or absence of comorbidities.
Abbreviations: COPD, chronic obstructive pulmonary disease; NAFLD, non-alcoholic fatty liver disease; PASI, psoriasis area and severity index; PsA, psoriatic arthritis.
